# The Effect of Ginger Juice Processing on the Chemical Profiles of *Rhizoma coptidis*

**DOI:** 10.3390/molecules23020380

**Published:** 2018-02-10

**Authors:** Chunyu Yang, Fengqian Guo, Chen Zang, Cui Li, Hui Cao, Baoxian Zhang

**Affiliations:** 1 Institute of Chinese Materia Medica, China Academy of Chinese Medical Sciences, Beijing 100700, China; yangchunyu314@163.com (C.Y.); Fengqianguo@126.com (F.G.); czang@icmm.ac.cn (C.Z.); licui198209@126.com (C.L.); 2 School of Pharmacy, Jinan University, Guangzhou 510632, China; kovhuicao@aliyun.com

**Keywords:** *Rhizoma coptidis*, processing, giner juice, alkaloid, volatiles

## Abstract

*Rhizoma coptidis* (RC) has been used as an herbal medicine in China for over one thousand years, and it was subjected to specific processing before use as materia medica. Processing is a pharmaceutical technique that aims to enhance the efficacy and/or reduce the toxicity of crude drugs according to traditional Chinese medicine theory. In this study, the chemical profiles of RC, ginger juice processed RC (GRC), and water processed RC (WRC) was determined to reveal the mechanism of processing of RC. UPLC-QTOF-MS analysis of methanol extract of RC, GRC, and WRC has been conducted to investigate the effect of processing on the composition of RC. HPLC-PDA was used to determine the variance of total alkaloids and seven alkaloids of RC during the processing. The volatiles of RC, GRC and ginger juice were separated by distillation, the change of volatiles content was recorded and analyzed, and the qualitative analysis of the volatiles was carried out using GC-MS. The microstructures of RC, GRC and WRC were observed using a light microscope. Results showed that ginger juice/water processing had limited influence on the composition of RC’s methanol extract, but significant influence on the content of some alkaloids in RC. Ginger juice processing significantly increased (*p* < 0.05) the volatiles content of RC and changed the volatiles composition obviously. Processing also had an influence on the microstructure of RC. This research comprehensively revealed the mechanism of ginger juice processing of RC.

## 1. Introduction

*Rhizoma coptidis* (RC) is the dried rhizome of Coptis chinensis Franch, Coptis deltoidea C. Y. Cheng et Hsiao or Coptis teeta Wall (Fam. Ranunculaceae) [1], the most common used name is Coptis chinensis Franch. RC has been used in China for thousands of years and according to traditional Chinese medicine theory, RC can “clear the damp-heat, purge the fire and counteract the poison” [1,2]. Recent studies have indicated that RC possesses multispectral therapeutic activities, including antibacterial, antifungal, antiviral, antihyperglycemia, antihyperlipidemia, antihypertension, anti-inflammatory, and anti-oxidation effects [3,4].

Processing of Chinese Materia Medica (CMM) is a pharmaceutical technique to fulfill the different requirements of therapy, dispensing and making preparations according to traditional Chinese medicine theory. The aims of processing are to enhance the efficacy and/or reduce the toxicity of crude drugs [5]. Improper processing of drugs was one of the main reasons of occurrence and influencing factors of TCM-induced diseases [6].

RC was subjected to specific processing before it was used as materia medica in China. The purposes of processing RC were to reduce the side effects in the gastrointestinal tract and enhance its efficacy. The Chinese pharmacopoeia recorded three types of processed RC, RC processed with wine, RC processed with ginger juice, and RC processed with Fructus Evodiae [1]. Processed RC using different methods demonstrate different properties, and their pharmacological effects in the clinic were also different. RC processed with ginger juice was used for the treatment of stomach issues and it also has an anti-vomiting effect [7].

**Figure 1:**
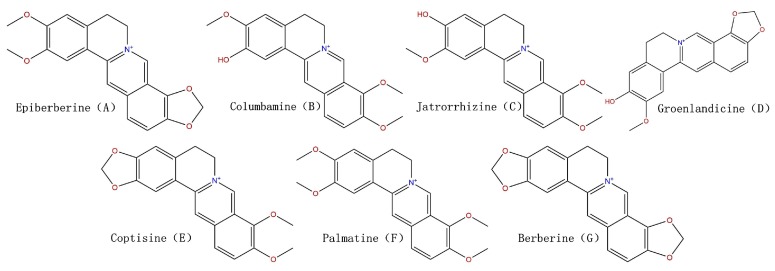
Chemical structure of the studied seven alkaloid in extracts of processed and unprocessed *Rhizoma coptidis*.

The major active constituents in Coptis chinensis Franch are alkaloids, including epiberberine, columbamine, jatrorrhizine, groenlandicine, coptisine, palmatine and berberine, shown in Figure 1. A number of studies on the determination of alkaloid content of RC have been reported [8,9,10]. However, little research on the difference of processed and unprocessed RC has been reported. Jiang et al. [7] identified 13 alkaloids in RC and processed RC by the UPLC-QTOF/MS method, and proved the chemical differences between crude and processed RC based on the PCA analysis. Huang et al. [11] established HPLC-PAD method for the determination of 11 Alkaloids of crude and wine-processed RC. Qian et al. [12] developed and validated a UHPLC-ESI-MS/MS method for simultaneous quantitative determination of ten alkaloids in rat plasma after administration of crude and wine-processed RC aqueous extracts.

In addition, even though most studies of the processing of RC focused on alkaloids, the influence of processing on RC was not only the changing of alkaloids, and further study on the change of other constituents was needed. Ginger juice was a commonly-used adjuvant for CMM processing, which contains active ingredients such as gingerols, shogaols and volatiles [13,14]. The effect of ginger’s main component on RC’s processing has not been reported before, and the mechanism of processing on RC was also not fully illuminated.

In this study, the chemical profiles of unprocessed RC, ginger juice-processed *Rhizoma coptidis* (GRC) and water processed *Rhizoma coptidis* (WRC) were investigated (WRC was used as a control group). Both alkaloids and volatiles of RC and processed RC were determined. The aim of this study was to investigate the effect of processing on RC and to reveal the mechanism of ginger juice-processing working on RC.

## 2. Results

### 2.1. The Effect of Processing on the Composition of Methanol Extract of Rhizoma coptidis

**Figure 2:**
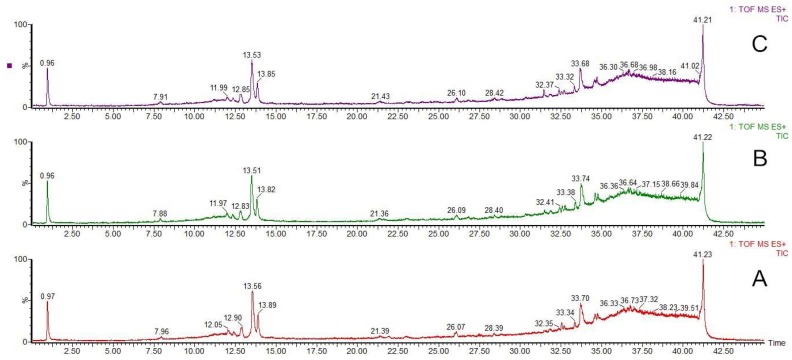
UPLC-QTOF-MS total ion current chromatogram of a methanol extract of *Rhizoma coptidis* (**A**), ginger juice-processed *Rhizoma coptidis* (**B**) and water-processed *Rhizoma coptidis* (**C**).

As shown in Figure 2, the chromatograms of RC, WRC and GRC are basically the same, and the results of the peak analysis revealed that no changes of the peaks were detected. It seems that ginger juice processing and water processing had no obvious influence on the composition of the methanol extract of RC. Wu et al. [15] reported that during the wine-processing of RC, a portion of berberubine was formed from berberine because of the high temperature. Jiang et al. [7] reported that the auxiliary material or heating process may enhance the transformation of dihydrochelerythrine from berberine during the processing of RC and the acidic condition in processing could enhance the reaction of protoberberine. However, in our research, no obvious chemical reactions were detected, which suggested that the reaction of berberine was not only influenced by temperature, but the auxiliary material (adjuvant for processing) also played an important role in the processing of RC.

Ginger juice containing methanol-soluble gingerols, and one of the main active ingredients, 6-gingerol was anticipated to be transferred into RC during ginger processing before the experiment. However, no gingerol or shogaol were detected in the methanol extract of GRC. A reasonable explanation was that the amounts of gingerols and shogaol transferred into RC were too low to be detected. Above all, methanol-soluble compounds transferring from the adjuvant to the RC was not the main mechanism of ginger juice processing.

### 2.2. The Effect of Processing on Alkaloid Content of Rhizoma coptidis

**Figure 3:**
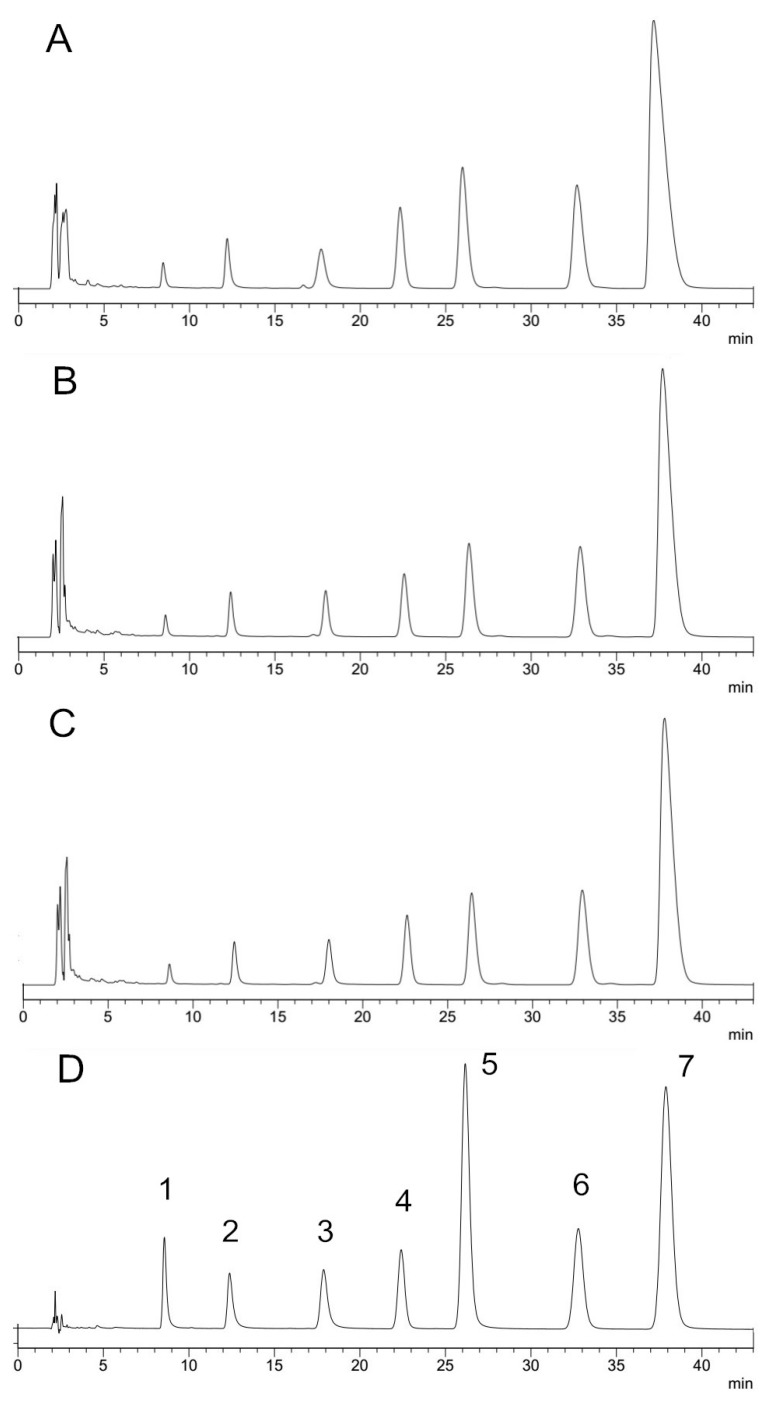
HPLC chromatogram of methanol extract of *Rhizoma coptidis* (**A**), ginger juice-processed *Rhizoma coptidis* (**B**), water-processed *Rhizoma coptidis* (**C**), and the mixture of reference substances (**D**). 1. Groenlandicine 2. Jatrorrhizine 3. Columbamine 4. Epiberberine 5. Coptisine 6. Palmatine 7. Berberine.

According to Wu et al. [15] processing may cause a quantitative change of alkaloid content. The main alkaloids of RC have been identified using reference substances by HPLC, the chromatograms are shown in Figure 3.

Quantitative study of total alkaloids and seven alkaloids of RC and processed RC (WRC, GRC) was conducted in this research. The results of the content determination of total alkaloids and seven alkaloids in RC, WRC, and GRC are listed in Table 1. Ginger juice processing increased the total alkaloid content (TAC) of RC from 11.99% ± 0.23% to 12.47% ± 0.46%, but the difference was not significant (*p* > 0.05). Water processing significantly (*p* < 0.05) decreased the total alkaloid content of RC to 10.73% ± 0.03%. As the RC specimens were subjected to covered moistening of equal time and drying at the same temperature during ginger juice processing and water processing, the different variation tendency of the TAC of RC processed by different methods suggested the chemical compositions of ginger juice was helpful to retain or increase the alkaloids of RC. Zhao et al. claim [5] that the wine could increase the dissolution of chemicals, and that vinegar may transfer the free alkaloids into soluble alkaloids, which would increase the alkaloid content. Ginger juice may also increase the alkaloid contents of RC by a similar mechanism.

**Figure 4:**
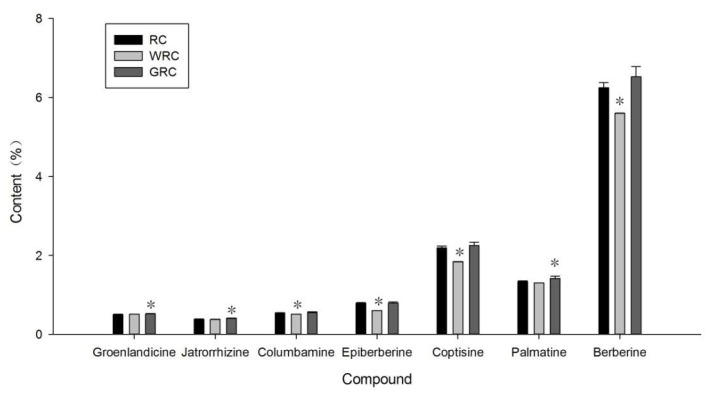
Seven alkaloids content of *Rhizoma coptidis* (RC), water-processed *Rhizoma coptidis* (WRC) and ginger juice-processed *Rhizoma coptidis* (GRC). Values are means ± SD, *n* = 3. * *p* < 0.05 of processed RC against RC.

As shown in Figure 4, ginger juice processing caused a significantly increase (*p* < 0.05) of groenlandicine, jatrorrhizine, and palmatine content of RC, while water processing led to a significant decrease (*p* < 0.05) of the columbamine, epiberberine, coptisine and berberine content of RC, while other compounds did not show significant difference. Previous studies showed that vinegar processing can improve the solubility of some alkaloids from Rhizoma corydalis and the mechanism was the formation of water-soluble salts [15]. The pH value of the ginger juice used in this study was determined to be about 6.5, and the weakly acidic condition was probably a factor that influenced the groenlandicine, jatrorrhizine, and palmatine content of RC.

The relative content of groenlandicine and jatrorrhizine in RC was not high, but the previous study showed groenlandicine and jatrorrhizine had a high number of pharmacological activities. Groenlandicine exhibited moderate inhibitory effects on anti-diabetic complications [16] and anti-Alzheimer effect [17], and it was also identified as an active principle with topoisomerase I-mediated DNA cleavage activity in vitro [18]. Jatrorrhizine exerted hepatoprotective activities [19], neuroprotective activity [20] and antihypercholesterolemic activity [21]. Palmatine had the third highest content of alkaloids in RC, and it was proven to have anti-bactericidal activity against *H. pylori* in vitro and in vivo [22], gastroprotective effect against gastric ulcers [23], heartprotective activity against acute myocardial injury [24] and memory-enhancing activity [25]. Jatrorhizine, groenlandicine, and palmatine exhibited significant peroxynitrite scavenging activities and ameliorated cell damage [17,26]. The increased of groenlandicine, jatrorrhizine, palmatine content in RC may relate to the change of RC pharmacologic activities.

### 2.3. The Effect of Ginger Juice Processing on Volatile Content of Rhizoma coptidis

The volatile content of RC and GRC was 0.003% ± 0.001% and 0.009% ± 0.001%, respectively. The volatile content of RC significantly increased (*p* < 0.05) after processing with ginger juice. Miyazawa et al. [27] reported dry coarsely-powdered RC (Coptis chinensis) hydro-distilled with a Likens–Nickerson-type apparatus using diethyl ether to yield 0.052% of yellowish green oil, but the author did not provide details of the separation method of the volatiles. The difference of the volatile content between previous research and our study may be attributed to the different method of volatiles extracting and the difference of the specimens. Additionally, no research on the quantitative change of volatiles of RC before and after ginger juice processing was reported before. The increase of volatile content of RC being processed with ginger juice was mainly caused by the transfer of volatiles from the ginger juice (see Section 2.4).

### 2.4. The Effect of Ginger Juice Processing on Volatiles Composition of Rhizoma coptidis

**Figure 5:**
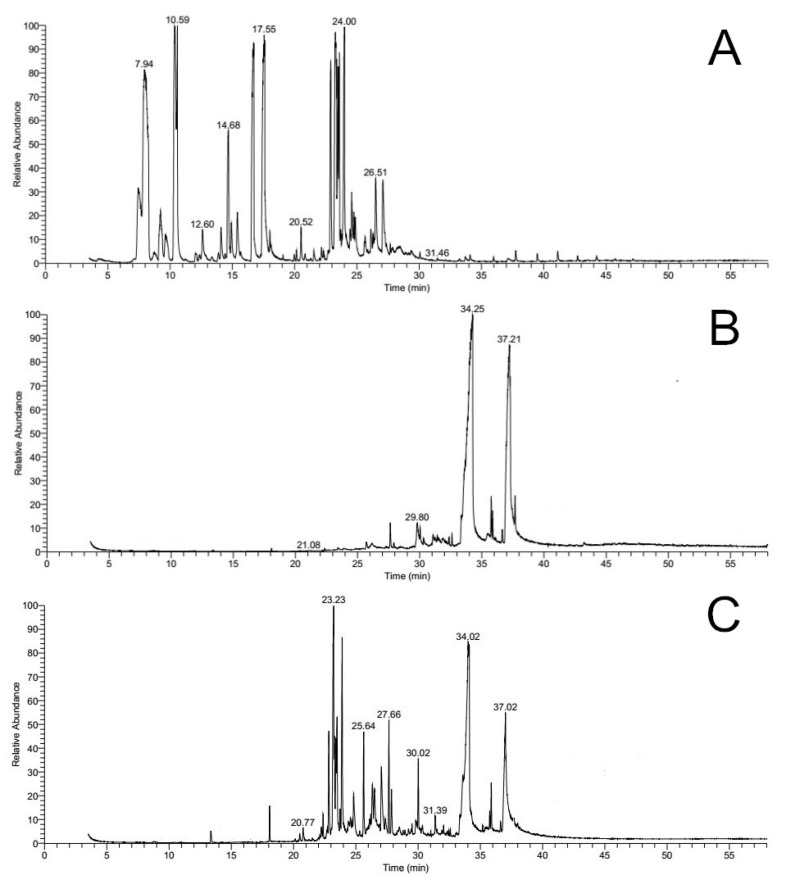
The total ion chromatogram of volatiles in ginger juice (**A**), *Rhizoma coptidis* (**B**), and ginger juice-processed *Rhizoma coptidis* (**C**) by GC-MS.

As shown in Figure 5, the volatiles of ginger juice were mainly compounds with relatively low boiling points (75 °C to 160 °C); the volatiles of RC and GRC were mainly compounds with the boiling point ranging from 180 °C to 235 °C and 135 °C to 235 °C, respectively. After ginger juice processing, the composition of the volatiles of RC obviously changed, and compounds with low boiling points (from the ginger juice) were transferred into RC (see Table 2). However, the volatiles of GRC were not all of the volatiles of RC and ginger juice. During the ginger juice processing, the heat volatilized most of the low boiling point volatiles (<135 °C) and part of the volatiles with low content in RC, while most volatiles with high boiling points (>135 °C) were retained.

As shown in Table 2, the composition of volatiles of RC, GRC and ginger juice were analyzed. The numbers of identified peaks of RC, GRC and ginger juice were 8, 19, and 38, accounting for 82.73%, 86.29%, and 94.64% of their total volatile content, respectively. The main constituents (>3%) of volatiles of ginger juice included camphene (14.85%), β-phellandrene (12.89%), endo-borneol (3.75%), neral (8.87%), geranial (10.31%), α-curcumene (4.32%), zingiberene (8.40%), α-farnesene (3.40%) and β-sesquiphellandrene (5.36%), and that was in accord with previous study [28,29]. Miyazawa et al. [27] reported the main constituents of the essential oil of RC were found to be m-acetylanisole, 2-methylpropanoic acid, 3-hydroxy-2,4,4-trimethylpentyl ester, 2-methyl-propanoic acid, 2,2-dimethyl-1-(2-hydroxy-1-methylethyl) propyl ester, and phenol. Gao et al. [30] claimed that monoterpenes and sesquiterpenes are major volatile compounds (volatiles were extracted using headspace solid-phase microextraction) in the coptis species. However, in our study, *cis*-9, *cis*-12-octadecadienoic acid (40.80%), n-hexadecanoic acid (34.00%) and tetradecanoic acid (3.17%) were determined to be the main constituents of RC volatiles. Different extracting methods of volatiles and different sources of RC specimens were the most likely reason causing the variance in the results. The volatile composition of RC obviously changed after processing with ginger juice, 13 new compounds were detected while two compounds (pentadecanal (0.14%), *cis*-10-heptadecenoic acid (0.75%)) were lost. The main constituents of GRC volatiles were α-curcumene (4.32%), zingiberene (15.73%), α-farnesene (4.03%), β-bisabolene (4.95%), β-sesquiphellandrene (9.28%), eudesm-4(14)-en-11-ol (4.93%), n-hexadecanoic acid (19.54%), and *cis*-9,*cis*-12-octadecadienoic acid (11.70%). Among them, n-hexadecanoic acid and *cis*-9,*cis*-12-octadecadienoic acid were the original compounds of RC, and the others were from ginger juice, it is worth mentioning that the relative content of n-hexadecanoic acid and *cis*-9,*cis*-12-octadecadienoic acid obviously increased after ginger processing(from 0.83:1 to 1.67:1). Additionally, nerolidol and β-bisabolol were detected in GRC volatiles but were detected in volatiles of RC or ginger juice. However, nerolidol and β-bisabolol were reported to be the constituent of ginger [29]. A reasonable explanation was the relative contents of nerolidol and β-bisabolol in volatiles of ginger juice were low and they were not identified, and that nerolidol and β-bisabolol in GRC were also from ginger juice.

The previous study showed ginger oil had strong antimicrobial activity, antioxidant activity, anti-inflammatory and antinociceptive properties, and the results of volatiles composition analysis indicated α-zingiberene, curcumene, sesquiphellandrene, citral, camphene, α-farnesene were the active ingredients [31,32,33,34]. Ginger oil also had antifungal activity [35], and ginger essential oil inhalation has implications for alleviating postoperative nausea and vomiting in abdominal surgery patients [36]. Additionally, zingiberene and sesquiphellandrene has anticancer potential [37,38]. Above all, the terpenes introduced from ginger juice in GRC during the processing may enhance the antimicrobial activity and antioxidant activity of RC and bring new pharmacological activities to RC.

### 2.5. The Effect of Processing on the Microstructure of Rhizoma coptidis

Zhao et al. [5] stated that sometimes the processing practice could increase the dissolving rate of chemicals which induces the change of chemical contents. In this research, the microstructures of RC and processed RC were observed using light microscope, and the authors attempted to reveal whether a relationship existed between the change of alkaloid content and the microstructure of RC during ginger juice processing.

**Figure 6:**
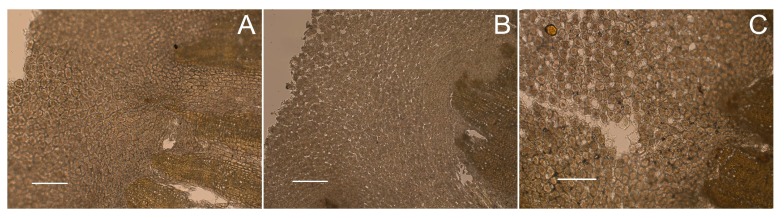
The microscopic image of *Rhizoma coptidis* (**A**), ginger juice processed-*Rhizoma coptidis* (**B**) and water-processed *Rhizoma coptidis* (**C**) slices. Scale bar: 50 μm.

As shown in Figure 6, the pith texture of unprocessed RC was very tight, and no obvious intercellular space of parenchyma cells existed. After processing with ginger juice/water, the pith texture of RC became much looser. A large number of fine cracks were observed in the pith part of GRC, and irregular intercellular space formed potential paths to transfer compounds outwards, which may be one of the mechanisms by which ginger processing increased the alkaloid content of the methanol extract of RC. The irregular intercellular space of parenchyma cell was also observed in the pith part of WRC, which suggests water infiltration and thermal damage caused the change of the pith texture of RC. Since ginger juice processing and water processing changed the microstructure of RC in the same way, and both the two processing methods had a significant influence (*p* < 0.05) on some alkaloid content in RC, it was reasonable to deduce that the change of RC alkaloid content during processing may be related to the change in the microstructure of RC.

## 3. Materials and Methods

### 3.1. Material and Regents

Reference substances of groenlandicine, jatrorrhizine hydrochloride, columbamine, epiberberine, coptisine hydrochloride, palmatine hydrochloride and berberine hydrochloride were purchased from Desite Biotechnology Co., Ltd. (Chengdu, Sichuan, China). HPLC-grade acetonitrile and methanol were obtained from Fisher chemical (Shanghai, China). All other reagents were of analytical grade.

*Rhizoma coptidis* (dried rhizomes of the *Coptis chinensis Franch*) was purchased from Anguo Changda Medicinal Material Company (Baoding, Hebei, China) and authenticated by Professor Hui Cao in Jinan University, China. Ginger juice was prepared in the lab using fresh matured gingers (*Zingiber officinale*) purchased from a Sichuan local ginger market, and the ratio of ginger to ginger juice was 1:1 (*m:v*).

### 3.2. Processing of Rhizoma coptidis

The processing of ginger juice processed *Rhizoma coptidis* (GRC) was in accordance with Chinese pharmacopoeia; dried slices were mixed well with ginger juice, in a pot, heated with a gentle fire until the ginger juice is completely absorbed, and the drugs removed and allow to cool. The ratio of ginger to *Rhizoma coptidis* was 12.5:100. Processing *Rhizoma coptidis* was also conducted using water instead of ginger juice, labeled as WRC.

### 3.3. Preparation of Rhizoma coptidis Methanol Extracts

An accurately-weighted 0.2 g powder of crude RC, GRC, or WRC, was transferred into a stopper conical flask, and 25 mL of methanol: hydrochloride (100:1) was accurately added and weighed respectively. The extract was ultrasonicated for 30 min and cooled to room temperature, then methanol: hydrochloride (100:1) was added to compensate for the lost weight. The solution was filtered through a 0.45 μm membrane before injection.

### 3.4. Preparation of Standard Solution

The reference substances of groenlandicine, jatrorrhizine hydrochloride, columbamine, epiberberine, coptisine hydrochloride, palmatine hydrochloride and berberine hydrochloride were accurately weighed and dissolved in methanol: hydrochloride (100:1) to make up the stock solutions. The mixed standard solution was prepared by dilutions of the stock solution with methanol: hydrochloride (100:1) to obtain a final mixed standard solution containing 0.008 mg·mL^−1^ groenlandicine, 0.0065 mg·mL^−1^ jatrorrhizine, 0.012 mg·mL^−1^ columbamine, 0.013 mg·mL^−1^ epiberberine, 0.046 mg·mL^−1^ coptisine, 0.019 mg·mL^−1^ palmatine and 0.0535 mg·mL^−1^ berberine.

### 3.5. Qualitative Analysis of Rhizoma coptidis Extraction by UPLC-QTOF-MS

All chromatographic peaks were identified and confirmed using UPLC-QTOF-MS experiment using an ACQUITY UPLC system (Waters, Milford, MA, USA) coupled to a Xevo™ G2 Q-Tof triple quadrupole mass spectrometer (Waters, Milford, MA, USA). Chromatographic separation was conducted using an Agilent SB-C18 column (4.6 mm × 250 mm i.d., 5 μm. Agilent, Santa Clara, CA, USA) at a flow rate of 0.3 mL/min. The mobile phases were methanol (A) and 0.1% formic acid aqueous solution (B) with gradient elution program listed in Table 3. The injection volume was 10 µL and the column temperature was 40 °C. MS spectra were recorded in the range of *m/z* 50–1200 using electrospray ionization (ESI) as the ionization source in positive/negative ion-switching mode. The mass spectrometer settings used were: capillary voltage: 3 kV, source temperature: 100 °C, desolvation temperature: 300 °C, cone gas (nitrogen) flow: 50 L·h^−1^, desolvation gas (nitrogen) flow: 990 L·h^−1^. Instrument control and data acquisition and evaluation were performed with the Micromass MassLynx 4.1 software package provided by Waters.

### 3.6. Quantitative Analysis of the Alkaloid Content of Rhizoma coptidis Extract by HPLC-PDA

The alkaloids of crude RC, WRC, and GRC were determined by the HPLC method [50]. A Shimadzu liquid chromatography system (LC-20AT, SPD-M20A, SIL-20A, CTO-10AS, Shimadzu Co., Kyoto, Japan) was employed to carry out the determination. Chromatographic separation was performed on an Agilent Zorbax Extend-C18 column (4.6 mm × 250 mm, 5 μm). The mobile phase was prepared from acetonitrile (A) and a mix solution (0.25 mol·L^−1^ ammonium acetate and 8 mmol·L^−1^ lauryl sodium sulfate, adjusted to pH 9.3 before use) (B) at the ratio 36:64. The flow rate was 1.0 mL·min^−1^, the injection volume was 10 μL, and the column temperature was maintained at 35 °C. The UV absorbance between 200 and 400 nm was detected, and the absorbance at 270 nm was used for calculation. The alkaloid content for each alkaloid was calculated by comparing the peak area with the standard curve. The total alkaloid content was calculated as the sum of the content of the 7 individual alkaloids.

### 3.7. Qualitative and Quantitative Analysis of Volatiles of Processed and Unprocessed Rhizoma coptis by GC-MS

The volatiles of crude RC and GRC were extracted by the water distillation method. A total of 500 g of RC/GRC was transferred to a 10,000 mL flask, 10 times the amount of water was then added to the flask and mixed well. A volatile oil extractor was employed to extract the volatiles, extracted for five hours after boiling. The volume of volatiles was recorded. The volatiles were collected, and then dehydrated with a proper amount of anhydrous sodium sulfate. Twenty microliters of dissolved volatiles with 2 mL n-hexane was used as the sample for qualitative analysis.

The qualitative analysis of volatiles was conducted on a Trace GC ULTRA gas chromatograph (Thermo Scientific, Waltham, MA, USA) directly coupled to an ISQ Single Quadruple MS (Thermo Scientific). The volatiles were separated using an Agilent J&W DB-5 column (30 m × 0.25 mm × 0.25 μm). The oven temperature program was as follows: 50 °C (held for 3 min), then raised to 300 °C at a rate of 5 °C/min (held for 5 min). Helium was used as the carrier gas at a flow rate of 1.0 mL/min. The MS fragmentation was performed by electronic impact (EI) at 70 eV, a source temperature of 300 °C, scanning times of 0.2 s, and a mass acquisition range of 35–800 amu. All compounds were also confirmed by the matching of their mass spectra with the NIST 2.0 mass spectral database (National Institute of Standards and Technology, Gaithersburg, MD, USA). Also, the retention indices were calculated for all volatile constituents using a homologous series of n-alkanes.

### 3.8. Observation of the Microstructure of Processed and Unprocessed Rhizoma coptidis

The crude RC, WRC, and GRC were soaked with 5% glycerin solution, then the microsection of crude RC, WRC, and GRC were prepared by freezing microtome (Leica CM1860, Nussloch, Germany). The microstructure of each microsection was observed using a light microscope (Axio Imager A2, Jena, Germany).

### 3.9. Statistical Analysis

The experiment was performed in triplicate. The experiment data was analyzed using SPSS 19.0 (Chicago, IL, USA) for analysis of variance (ANOVA) and Duncan’s test (*p* < 0.05). The data were reported as the mean ± standard deviation (SD).

## 4. Conclusions

Ginger juice processing is a very important technique to enhance the efficacy and reduce the toxicity of crude drugs. However, to date, the research on ginger juice processing has been limited, and the effect of ginger juice processing on crude drugs was still unclear. This research comprehensively revealed the effect of ginger juice processing on the chemical profiles of RC.

Generally, ginger juice/water processing did not obviously change the composition of the methanol extract of RC. Instead, ginger juice processing kept the TAC of RC with no significant change and significantly increased (*p* < 0.05) the content of three alkaloids; water processing significantly decreased (*p* < 0.05) the TAC and the content of four alkaloids in RC. As an adjuvant for processing, ginger juice helped to retain the main active ingredient of RC, alkaloids during the processing. The mechanism of ginger juice processing increased some alkaloid content of RC and may relate to the microstructure change caused by the processing, but further studies will be done to illuminate the relationship between microstructure change and alkaloid content change in RC.

In addition, ginger juice processing significantly increased (*p* < 0.05) the volatile content of RC; after ginger juice processing, the composition of RC volatiles also obviously changed, 13 new compounds were introduced, and two original compounds gone. The new compounds of RC volatiles were mainly from the ginger juice. In order to investigate the influence of the changing of RC volatiles on pharmacological activities, the difference of the effect on the gastrointestinal tract between RC and processed RC will be tested in the near future. Also, the relationship between the change of the chemical profile and the pharmacological activity of RC will be investigated.

## Figures and Tables

**Table 1:** Alkaloid content of unprocessed and processed *Rhizoma coptidis*. RC: *Rhizoma coptidis*, GRC: ginger processed *Rhizoma coptidis*, WRC:water processed *Rhizoma coptidis*. For each column, values followed by the same letter did not share significant differences at *p* < 0.05 (Duncan’s test).

	Content of alka%loids (%)							
	Groenlandicine	Jatrorrhizine	Columbamine	Epiberberine	Coptisine	Palmatine	Berberine	Total
RC	0.50 ± 0.01a	0.38 ± 0.01a	0.54 ± 0.01a	0.79 ± 0.01a	2.19 ± 0.05a	1.33 ± 0.03a	6.25 ± 0.13a	11.99 ± 0.23a
WRC	0.51 ± 0.01a	0.38 ± 0.00a	0.51 ± 0.01b	0.60 ± 0.01b	1.84 ± 0.01b	1.30 ± 0.00a	5.6 ± 0.01b	10.73 ± 0.03b
GRC	0.52 ± 0.00b	0.41 ± 0.01b	0.55 ± 0.02a	0.79 ± 0.03a	2.25 ± 0.09a	1.42 ± 0.05b	6.53 ± 0.25a	12.47 ± 0.46a

**Table 2:** Volatiles compositions of RC, GRC and ginger juice.

Peak Number	RT	RIa	RIb	Identified Compounds	RC	GRC	Ginger Juice
1	4.28	800	800 [39]	Hexanal	-	-	0.19%
2	7.09	924	925 [40]	Tricyclene	-	-	0.05%
3	7.45	935	939 [39]	α-Pinene	-	-	2.91%
4	8.00	952	953 [39]	Camphene	-	-	14.85%
5	9.24	989	980 [39]	β-Pinene	-	-	2.32%
6	9.64	1001	1003 [39]	α-Phellandrene	-	-	1.23%
7	10.38	1025	1018 [39]	β-Phellandrene	-	-	12.89%
8	12.04	1079	1088 [39]	p-Mentha-1,4(8)-diene	-	-	0.32%
9	12.61	1097	1098 [39]	β-Linalool	-	-	0.91%
10	13.35	1122	1126 [41]	*cis*-p-Menth-2-en-1-ol	-	-	0.15%
11	14.09	1148	1148 [40]	Citronellal	-	-	0.86%
12	14.42	1159	1138 [42]	*cis*-Verbenol	-	-	0.07%
13	14.68	1168	1165 [39]	endo-Borneol	-	-	3.75%
14	14.92	1177	1183 [40]	Terpinen-4-ol	-	-	0.89%
15	15.40	1193	1189 [39]	α-Terpineol	-	-	1.39%
16	16.70	1241	1228 [40]	Neral	-	-	8.87%
17	17.55	1272	1270 [39]	Geranial	-	-	10.31%
18	18.01	1289	1291 [40]	2-Undecanone	-	-	0.30%
19	19.06	1328	1337 [43]	δ-Elemene	-	-	0.06%
20	19.97	1362	1374 [40]	Cyclosativene	-	-	0.10%
21	20.23	1371	1377 [39]	α-Copaene	-	-	0.16%
22	20.52	1382	1391 [39]	β-Elemene	-	0.34%	0.30%
23	21.29	1413	1418 [39]	Caryophyllene	-	-	0.04%
24	21.52	1424	1433 [43]	γ-Elemene	-	0.16%	0.22%
25	22.15	1452	1458 [39]	β-Farnesene	-	-	0.21%
26	22.83	1483	1486 [40]	α-Curcumene	-	4.27%	4.32%
27	23.22	1501	1502 [40]	Zingiberene	-	15.73%	8.40%
28	23.41	1507	1509 [39]	α-Farnesene	-	4.03%	3.40%
29	23.49	1510	1509 [39]	β-Bisabolene	-	4.95%	2.59%
30	23.71	1518	1513 [39]	γ-Cadinene	-	0.73%	-
31	23.90	1525	1532 [40]	β-Sesquiphellandrene	-	9.28%	5.36%
32	24.57	1548	1549 [43]	Elemol	-	0.94%	1.18%
33	24.83	1557	1564 [39]	Nerolidol	-	2.99%	0.85%
34	25.65	1586	1543 [44]	*cis*-Sesquisabinene hydrate	-	-	0.56%
35	25.86	1593	1586 [44]	*trans*-Sesquisabinene hydrate	-	-	0.07%
36	26.30	1609	1621 [41]	γ-Eudesmol	-	2.52%	0.19%
37	26.51	1616	1613 [45]	Isoaromadendrene epoxide	-	-	1.87%
38	27.06	1655	1649 [39]	Eudesm-4(14)-en-11-ol	-	4.93%	2.45%
39	27.37	1673	1681 [41]	β-Bisabolol	-	1.01%	-
40	28.44	1724	1715 [46]	Pentadecanal	0.14%	-	-
41	29.80	1782	1763 [47]	Tetradecanoic acid	3.17%	1.01%	-
42	32.59	1921	1926 [47]	Hexadecanoic acid, methyl ester	0.54%	0.23%	-
43	33.23	1947	1946 [48]	Geranyl-p-cymene	-	-	0.05%
44	33.56	1960	1951 [49]	Palmitoleic acid	1.31%	1.24%	-
45	34.25	1987	1983 [47]	n-Hexadecanoic acid	34.00%	19.54%	
46	35.48	2069	-	*cis*-10-Heptadecenoic acid	0.75%	-	-
47	35.75	2090	2094 [47]	Linoleic acid, methyl ester	2.02%	0.69%	-
48	37.21	2173	2159 [47]	*cis*-9,*cis*-12-Octadecadienoic acid	40.80%	11.70%	-
Total					82.73%	86.29%	94.64%

RIa: Retention index calculated from retention times relative to that of n-alkanes (C6–C26) on the the DB-5 column. RIb: literature retention index. RC: *Rhizoma coptidis*, GRC: ginger juice-processed *Rhizoma coptidis*. “-” means not detected or not available.

**Table 3:** Gradient table of the UPLC elution program.

	Time (Min)	Flow Rate	%A	%B
**1**	Initial	0.3	10	90
**2**	3	0.3	10	90
**3**	35	0.3	100	0
**4**	40	0.3	100	0
**5**	40.1	0.3	10	90
**6**	45	0.3	10	90
